# Benzoyl ester formation in *Aspergillus ustus* by hijacking the polyketide acyl intermediates with alcohols

**DOI:** 10.1007/s00203-021-02182-0

**Published:** 2021-01-22

**Authors:** Liujuan Zheng, Shu-Ming Li

**Affiliations:** Institut für Pharmazeutische Biologie und Biotechnologie, Fachbereich Pharmazie, Philipps-Universität Marburg, Robert-Koch Straße 4, 35037 Marburg, Germany

**Keywords:** Benzoyl esters, Biosynthesis, *Aspergillus ustus*, Alcohols feeding, Polyketides

## Abstract

Accumulation of two benzoyl esters in *Aspergillus ustus* after feeding with alcohols was reported 30 years ago. To the best of our knowledge, the biosynthesis for these esters has not been elucidated prior to this study. Here, we demonstrate that these compounds are artifical products of the phenethyl polyketide ustethylin A biosynthestic pathway. In addition, four aditional benzoyl esters with different methylation levels were also isolated and identified as shunt products. Feeding experiments provided evidence that the enzyme-bound polyketide acyl intermediates are hijacked by externally fed MeOH or EtOH, leading to the formation of the benzoyl esters.

**Supplementary Information:**

The online version contains supplementary material available at 10.1007/s00203-021-02182-0.

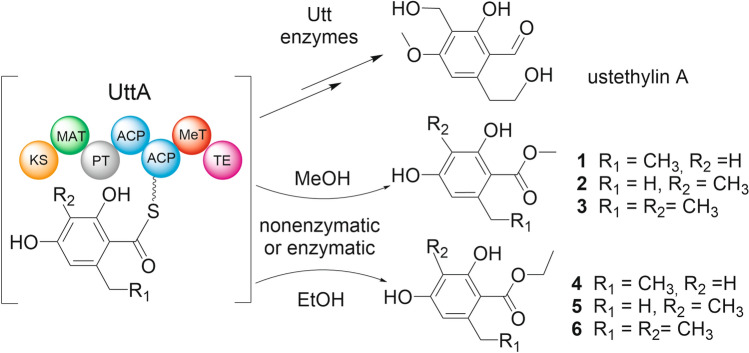

## Introduction

Secondary metabolites play an important role in ecological fitness of microorganisms, such as for protection from UV damage, toxic natural products and other microorganisms (Keller 2019). Polyketides with diverse biological and pharmacological activities are the most abundant fungal natural products (Keller et al. 2005; Ran and Li 2020). These compounds are biosynthesized by the well-studied multidomain proteins—polyketide synthases (PKSs). Based on the reduction status of their products, fungal PKSs can basically be divided into three class: nonreducing, partially reducing and highly reducing PKSs (Cox 2007). The typical nonreducing PKSs contain starter unit ACP transacylase (SAT), ketosynthase (KS), malonyl-CoA–ACP transacylase (MAT), product template (PT), acyl-carrier protein (ACP) and thioesterase (TE) domains (Crawford and Townsend 2010).

A study in 1987 reported the accumulation of 2,4-dihydroxy-3-methyl-6-ethyl benzoyl methyl and ethyl ester after feeding an *Aspergillus ustus* culture with MeOH and EtOH, respectively. Isotope incorporation was observed for both methyl groups of the aryl acid after feeding with [methyl-^13^C]-l-methionine, indicating their origin from methylation. Isotope labeling experiments also proved that other carbons are derived from acetate (De Jesus et al. 1987). However, no further biosynthetic studies have been reported for these compounds.

**Scheme 1.**
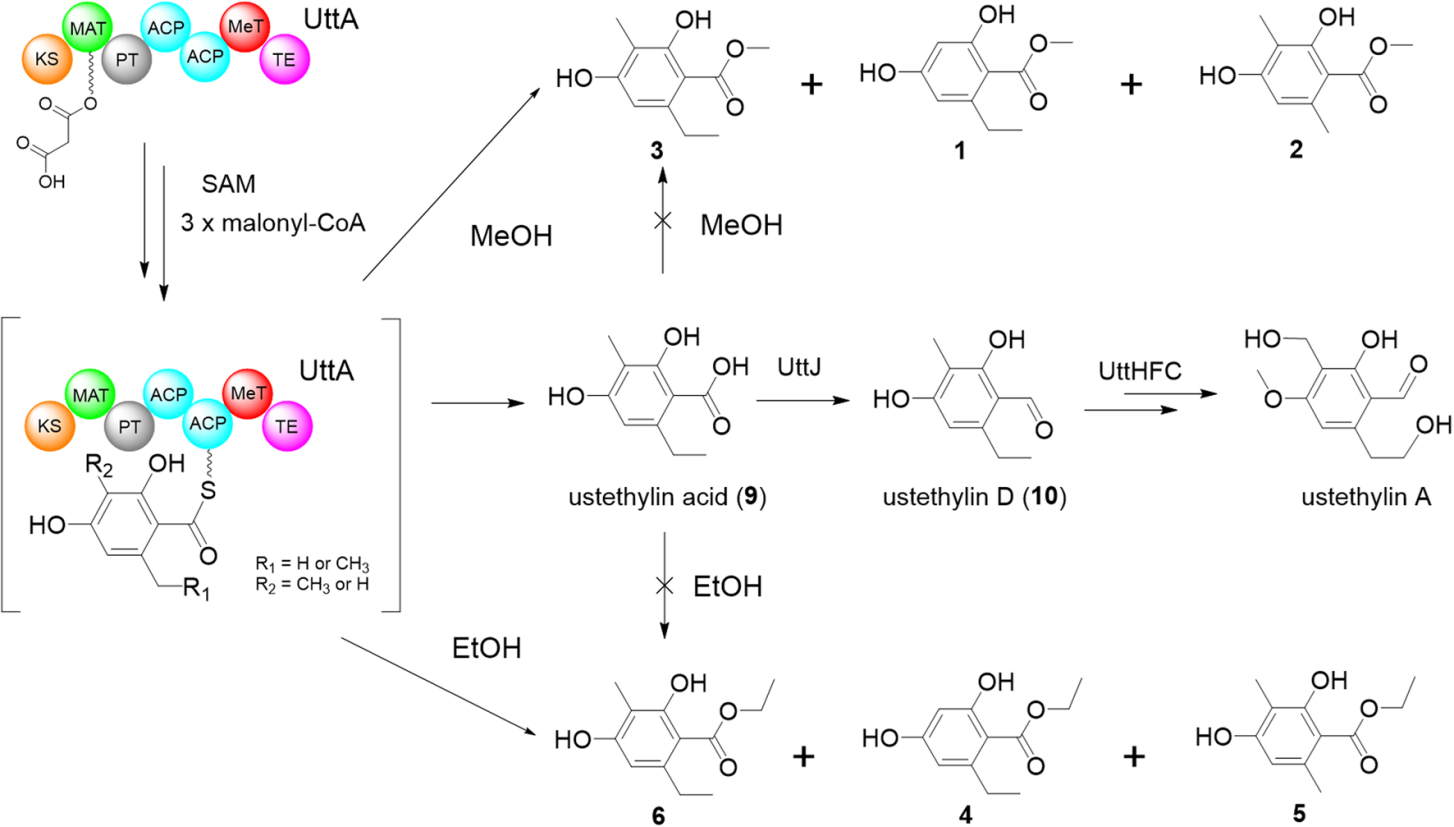
Simplified biosynthetic pathway of ustethylin A with **1**–**6** as shunt products after alcoholic feeding

**Fig. 1:**
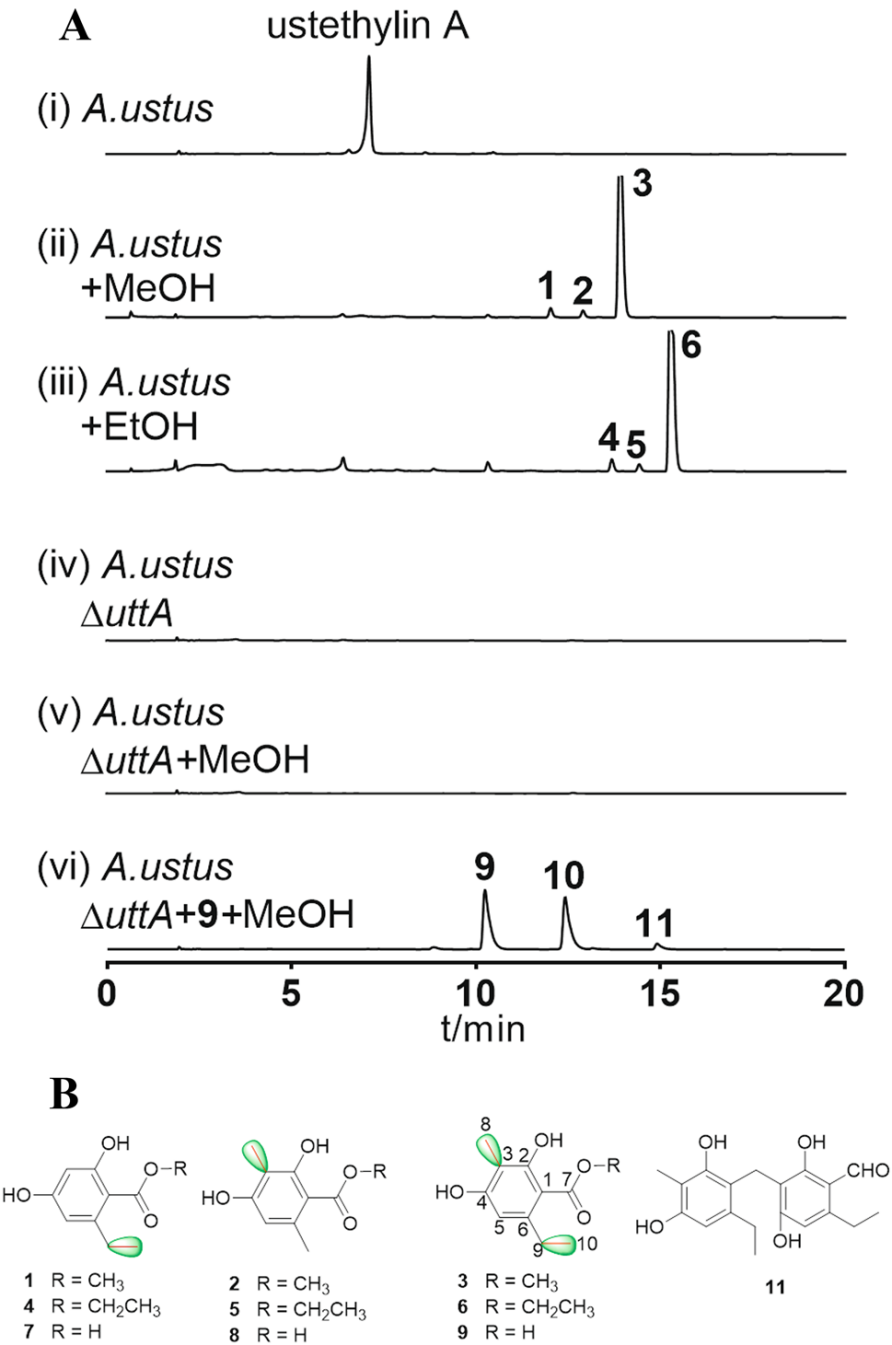
**a** HPLC analysis of the *A. ustus* extracts. UV detection was carried on a photodiode array detector and absorptions at 254 nm are illustrated. **b** Structures of **1–9** and **11**

Phenethyl-containing compounds are common microbial metabolites. In fungi, the ethyl groups in phenethyl residue are derived from *S*-adenosyl l-methionine and usually catalyzed by the methyltransferase domain of polyketide synthase (Zheng et al. 2020). Recently, we elucidated the first biosynthetic pathway of fungal phenethyl derivatives, i.e., that of ustethylin A in *A. ustus* 3.3904 (Zheng et al. 2020), a rare human pathogen fungus (Pi et al. 2015). In this pathway, the nonreducing PKS UttA is responsible for the formation of the key intermediate phenethyl benzoic acid. After reduction of the aryl carboxyl group to aldehyde by the NRPS-like enzyme UttJ, the methyl and ethyl groups are oxidized by the nonheme Fe^II^/2-oxoglutarate-dependent oxygenase UttH and the cytochrome P_450_ enzyme UttC, respectively. Phenolic methylation is catalyzed by the methyltransferase UttF (Scheme 1). The final pathway product ustethylin A was detected as the predominantly accumulated metabolite (Fig. 1a).

## Materials and methods

### Strains, media and growth conditions

*Aspergillus ustus* 3.3904 was purchased from China General Microbiological Culture Collection Center (Beijing, China) and cultivated in PDB (potato dextrose broth, Sigma) medium at 230 rpm and 30 °C for secondary metabolite production. Construction of the *A. ustus ΔuttA* mutant has been reported previously (Zheng et al. 2020).

### Alcoholic feeding experiments

1.5 mL (3%, v/v) of MeOH, EtOH or CD_3_OD were added into the three day-old *A. ustus* cultures in 250 mL flask containing 50 mL PDB medium, which were further maintained at 230 rpm and 30 °C for five days. 1 mL culture was extracted with EtOAc for LC–MS analysis.

For large-scale fermentation, 15 mL of MeOH or EtOH were added to 500 mL PDB medium in 2 L flask and cultivated under the same condition as mentioned above.

### Precursor feeding in *∆uttA* mutant

Compound **9** was dissolved in MeOH at a concentration of 18 mg/mL. 277 μL (5 mg) of this solution were added into the culture of the *A. ustus ∆uttA* mutant in 250 mL flask containing 50 mL PDB medium after fermentation at 230 rpm and 30 °C for three days. Two days later, 1 mL culture was extracted with EtOAc for LC–MS analysis.

### HPLC analysis and isolation

Extracts were analyzed on an Agilent HPLC series 1200 (Agilent Technologies) equipped with an Agilent Eclipse XDB-C18 column (5 μm, 4.6 × 150 mm). A linear gradient from 10 to 90% ACN in H_2_O in 20 min was used. The column was then washed with 100% ACN for 5 min and equilibrated with 10% ACN in H_2_O for another 5 min. Detection was carried out on a photodiode array detector and absorptions at 254 nm are illustrated in this study.

A semi-preparative Multospher 120 RP-18 column (5 μm, 10 × 250 mm) was used for isolation of the products for structural elucidation on the same HPLC system with the same solvents at a flow rate of 2 mL/min. Separation was done by isocratic elution with 25–70% ACN for 10–20 min.

### Large-scale fermentation, extraction and isolation of secondary metabolites

For isolation of compounds **1**–**3**, large-scale fermentation of *A. ustus* 3.3904 with MeOH was carried out as described above. The supernatant was separated from mycelia by filtration and extracted with equal volume of EtOAc for three times. The mycelia were extracted with acetone and concentrated under reduced pressure to afford an aqueous solution and then extracted with EtOAc for three times. Both EtOAc extracts were evaporated under reduced pressure to afford the crude extracts for further purification. The crude extract was separated on a silica gel column with CHCl_3_/MeOH (100:0–0:100) as elution solvents to give 8 fractions. Fraction 2 was purified on a semi-preparative HPLC (ACN/H_2_O), leading to 8.0 mg of **1**, 8.0 mg of **2** and 60.0 mg of **3**. Similar procedure was used for product isolation after EtOH feeding. 6.5 mg of **4**, 5.2 mg of **5** and 30.0 mg of **6** were obtained in high purity.

### LC–MS and MS analysis

Extracts were also analyzed on an Agilent HPLC 1260 series system equipped with a Bruker microTOF QIII mass spectrometer using an Agilent Eclipse XDB C18 column (5 μm, 4.6 × 150 mm). Separation was performed at a flow rate of 0.5 mL/min with a 40 min linear gradient from 5 to 100% ACN in H_2_O, both containing 0.1% (v/v) HCOOH. The column was then washed with 100% ACN for 5 min and equilibrated for 5 min with 5% ACN in H_2_O. The parameters of the spectrometer were set as the following: electrospray positive ion mode for ionization, capillary voltage with 4.5 kV, collision energy with 8.0 eV. Sodium formate was used in each run for mass calibration. The masses were scanned in the range of *m/z* 100–1500. Data were evaluated with the Compass DataAnalysis 4.2 software (Bruker Daltonik, Bremen, Germany).

### NMR analysis

NMR spectra of the isolated products were recorded at room temperature on a JEOL ECA-500 (JEOL, Akishima, Tokyo, Japan). The samples were dissolved in CDCl_3_ or CD_3_OD. All spectra were processed with MestReNov.9.0.0 (Mestrelab Research, Santiago de Compostella, Spain).

### Physiochemical properties of the compounds described in this study

**1:** white needle crystal; ^1^H NMR data see Table S1; HRMS (ESI) *m/z:* [M + H]^+^ calcd. for C_10_H_13_O_4_ 197.0808; found 197.0756.

**2:** white needle crystal; ^1^H NMR data see Table S1; HRMS (ESI) *m/z:* [M + H]^+^ calcd. for C_10_H_13_O_4_ 197.0808; found 197.0755.

**3:** white bulk crystal; ^1^H NMR data see Table S1, ^13^C NMR data see Table S2; HRMS (ESI) *m/z:* [M + H]^+^ calcd. for C_11_H_15_O_4_ 211.0965; found 211.0916.

**4:** white needle crystal; ^1^H NMR data see Table S1; HRMS (ESI) *m/z*: [M + H]^+^ calcd. for C_11_H_15_O_4_ 211.0965; found 211.0964.

**5:** white needle crystal; ^1^H NMR data see Table S1; HRMS (ESI) *m/z*: [M + H]^+^ calcd. for C_11_H_15_O_4_ 211.0965; found 211.0960.

**6:** white bulk crystal; ^1^H NMR data see Table S1; HRMS (ESI) *m/z:* [M + H]^+^ calcd. for C_12_H_17_O_4_ 225.1121; found 225.1119.

### Structural elucidation

The structures of the isolated products were elucidated by interpretation of their MS and NMR spectra (Figures S1–S9) and by comparison of these data with those described in the literature. **1** (Sher and Langer 2008), **2** (Schleich et al. 2006) **3** (De Jesus et al. 1987), **4** (Schleich et al. 2006), **5** (Sher and Langer 2008) and **6** (De Jesus et al. 1987) were identified as known compounds.

## Results and discussion

**Fig. 2:**
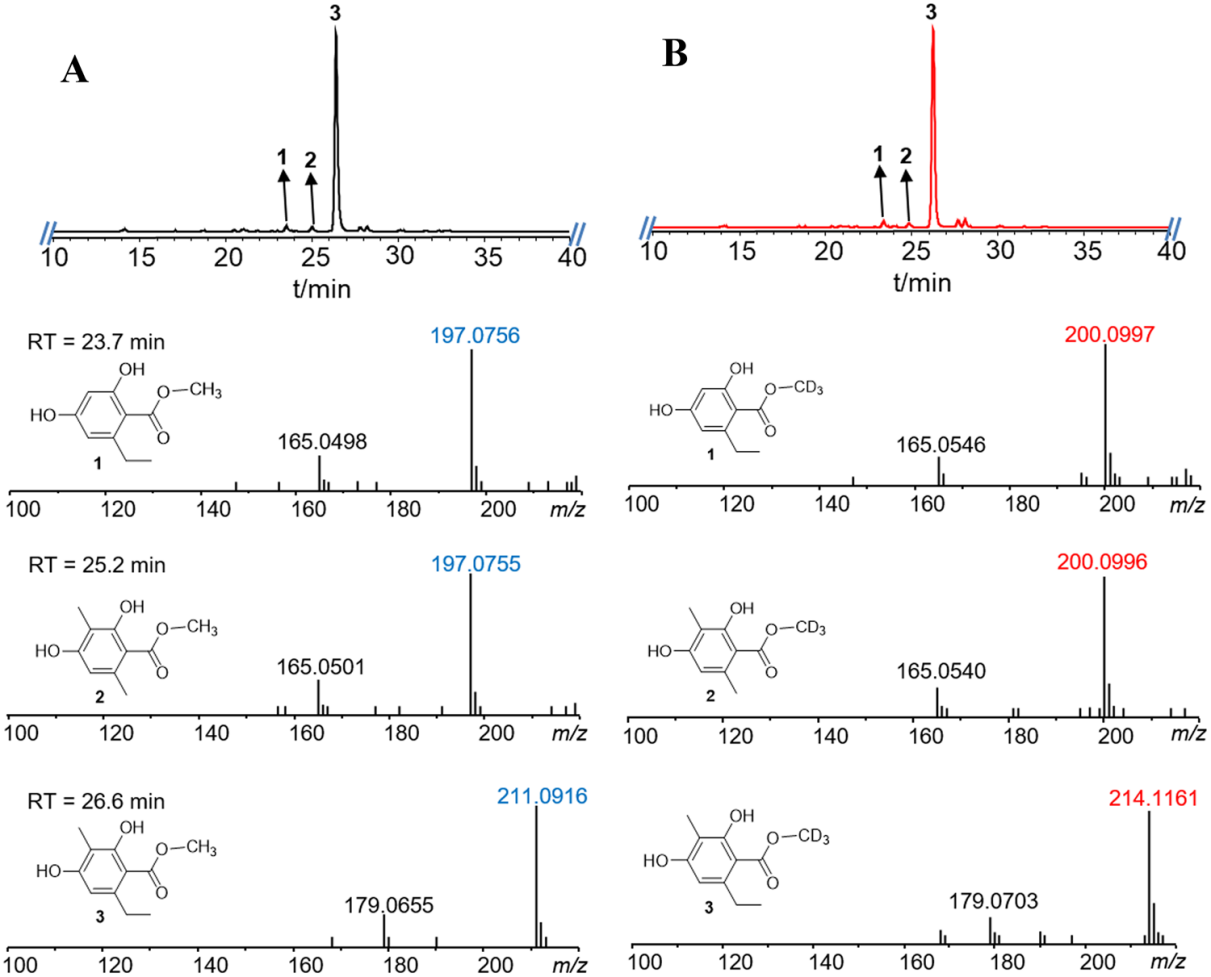
LC–MS results (positive mode) of *A. ustus* extracts after feeding with MeOH (**a**) and CD_3_OD (**b**). UV absorptions at 254 nm are illustrated

The aryl acids involved in the biosynthesis of ustethylins are the acyl components of the previously identified esters (De Jesus et al. 1987) (Fig. 1b). We speculated therefore that they are also derived from the ustethylin pathway. To prove our hypothesis, we repeated the feeding experiments with alcohols using three day-old cultures of *A. ustus* 3.3904 wild-type and an *uttA* deficient mutant in PDB medium. As shown in Fig. 1a, HPLC analysis of the EtOAc extract of the wild-type revealed the presence of ustethylin A as the predominant metabolite (Fig. 1a), as reported previously (Zheng et al. 2020). In the extract of the culture with externally fed MeOH and EtOH, three metabolites each **1**–**3** and **4**–**6** were detected, respectively. **3** as a major peak was accompanied by two minor ones **1** and **2** in the culture with MeOH. LC–MS analysis revealed that **3** has an additional methyl group than **1** and **2** (Fig. 2a). It is noteworthy that compounds **1**, **2**, **4** and **5** were not reported in the previous study (De Jesus et al. 1987).

For structural elucidation, **1** – **3** were isolated from a 2 L *A. ustus* culture after feeding with 60 mL MeOH. Comparison of the NMR data of the isolated products (Tables S1 and S2, Figures S1–S6) with those described in the literature confirmed **3** to be 2,4-dihydroxy-3-methyl-6-ethyl benzoyl methyl ester (De Jesus et al. 1987). NMR analysis also proved that **1** and **2** are two congeners of **3** lacking the *C3*-methyl group and replacing the *C6*-ethyl by a methyl group, respectively (Fig. 1b) (De Jesus et al. 1987; Schleich et al. 2006; Sher and Langer 2008). In analogy, the major peak **6** and the two minor ones **4** and **5** were also isolated from the culture fed with EtOH and identified as ethyl esters of the corresponding aryl acids (De Jesus et al. 1987; Schleich et al. 2006; Sher and Langer 2008) (Fig. 1b, Table S1, Figures S7–S9).

The isotopic feeding experiments in the previous study proved that the *O*-methyl group in compound **3** is not derived from methionine (De Jesus et al. 1987). The *O*-methyl groups in **1**–**3** originate likely from MeOH. To confirm this, CD_3_OD was fed into the *Aspergillus ustus* culture and the EtOAc extract was analyzed via HPLC–MS. Detection of [M + H]^+^ ion of **3** at *m/z* 214.1161 after feeding with CD_3_OD, 3 Daltons larger than that after feeding with MeOH at *m/z* 211.0916 (Fig. 2), proved the incorporation of the CD_3_ group into the structure of **3**. Similar MS pattern changes were also observed for compounds **1** and **2** (Fig. 2).

As reported previously (Zheng et al. 2020), the PKS UttA from the ustethylin biosynthetic pathway is responsible for the formation of the aryl acids **7**–**9** (Fig. 1b), the acyl moieties of **1**–**3** and **4**–**6**. To prove its function, *uttA* was replaced with a hygromycin B resistance cassette in our former study (Zheng et al. 2020). HPLC analysis of the culture extract of the Δ*uttA* mutant revealed the abolishment of ustethylin A production. Feeding this Δ*uttA* mutant with MeOH did not lead to an accumulation of **1**–**3** (Fig. 1a). Similar results were also observed after feeding the Δ*uttA* mutant with EtOH (data not shown). This proved unequivocally the involvement of UttA in the formation of **1**–**6**.

To figure out whether the ester formation is from free acids in *A. ustus*, **9** in MeOH was fed to the culture of Δ*uttA* mutant. As shown in Fig. 1a, no trace of **3** was detected. Instead, **9** was converted to the corresponding benzaldehyde **10** (ustethylin D) and a dimeric metabolite **11**, as described previously (Zheng et al. 2020). In addition, **3** was not detected after incubation of **9** with MeOH at 37 °C for 16 h (data not shown). These results indicated that the ester formation in **1**–**6** requires activation of the acidic precursors.

In the previous study, we proposed that the PKS UttA utilizes malonyl-CoA as both starter and extension unit. Two methylation steps, at C-3 of the benzene ring and the *C6*-methyl group, are involved in the formation of the aryl acyl-ACP molecules (Scheme 1), which are then released as free aryl acids and modified by tailoring enzymes. Our results in this study provide evidence that after the phenyl ring formation catalyzed by UttA, the acyl-ACP molecules can be hijacked by alcohols for ester formation, as depicted in Scheme 1. The formation of the minor products **1** and **2** after feeding with MeOH as well as **4** and **5** after feeding with EtOH confirmed the incomplete methylation steps by UttA, as observed in the previous study (Zheng et al. 2020). In vivo assays in our aforementioned study verified that reduction of the acid **9** to the benzaldehyde **10** by the NRPS-like enzyme UttJ is a prerequisite for further modification (Zheng et al. 2020). Therefore, the esters **1**–**6** cannot be further metabolized by the tailoring enzymes of the Utt pathway and were accumulated as artificial products.

Feeding n-PrOH to the culture of *A. ustus* wild-type led to the detection of trace amount of the corresponding ester by HPLC–MS analysis, which could not be isolated in substantial amount for structural identification. No ester formation was observed after feeding with i-PrOH or n-BuOH (data not shown). These results indicated that the ester formation is preferred for small alcohols. Ester bonds are commonly present in natural products and are usually formed during chain release in the polyketide (Wang et al. 2009) or nonribosomal peptide biosynthesis and by Baeyer–Villiger monooxygenases (Tsakos et al. 2015). However, spontaneous ester formation has also been described for carboxylic acids by incubation in alcoholic solvents at room temperature (Fan et al. 2020). Therefore, it cannot be concluded whether the formation of **1**–**6** is an enzymatic or nonenzymatic event (Scheme 1).

## Conclusions

In conclusion, we demonstrated in this study that the aryl acyl esters from *A. ustus* 3.3904 are shunt products of the ustethylin biosynthetic pathway as response for the externally fed alcohols. It will be interesting to investigate the physiological relevance of this response.

## Supplementary Information

Below is the link to the electronic supplementary material.

Supplementary file1 (PDF 583 KB)
