# Supplementary material for: Benzoyl ester formation in Aspergillus ustus by hijacking the polyketide acyl intermediates with alcohols
Source: Arch Microbiol. 2021 Jan 22;203(4):1795–800. doi: 10.1007/s00203-021-02182-0 (PMC8055633; doi:10.1007/s00203-021-02182-0)
Supplement: Supplementary file 1 — Supplementary file1 (PDF 583 KB) [file 203_2021_2182_MOESM1_ESM.pdf]

Supporting Information

Benzoyl ester formation in *Aspergillus ustus* by hijacking the polyketide acyl intermediates with alcohols

Liujuan Zheng[1] and Shu-Ming Li[1*]

Corresponding Author: Shu-Ming Li, E-Mail: shuming.li@staff.uni-marburg.de

1. Institut für Pharmazeutische Biologie und Biotechnologie, Fachbereich Pharmazie, Philipps-Universität Marburg, Robert-Koch Straße 4, 35037 Marburg (Germany)

Table of Contents

Table S1. ^{1}H NMR data of compounds **1** – **6** (500 MHz) 3

Table S2. ^{13}C NMR data of **3** (125 MHz in $CDCl_3$)..........4

Figure S1. ^{1}H NMR spectrum of compound **1** in CD_3OD (500 MHz)..........5

Figure S2. ^{1}H NMR spectrum of compound **2** in $CDCl_3$ (500 MHz)..........5

Figure S3. ^{1}H NMR spectrum of compound **3** in $CDCl_3$ (500 MHz)..........6

Figure S4. ^{13}C{^{1}H} NMR spectrum of compound **3** in $CDCl_3$ (125 MHz) 6

Figure S5. HSQC spectrum of compound **3** in $CDCl_3$ 7

Figure S6. HMBC spectrum of compound **3** in $CDCl_3$..........7

Figure S7. ^{1}H NMR spectrum of compound **4** in $CDCl_3$ (500 MHz)..........8

Figure S8. ^{1}H NMR spectrum of compound **5** in $CDCl_3$ (500 MHz)..........8

Figure S9. ^{1}H NMR spectrum of compound **6** in $CDCl_3$ (500 MHz)..........9

Table S1. ^{1}H NMR data of compounds **1 – 6** (500 MHz)

Compounds	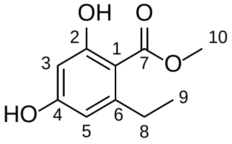 **1 (**CD_3OD**)**	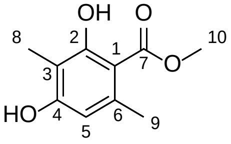 **2 (**$CDCl_3$**)**	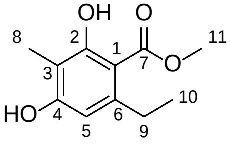 **3 (**$CDCl_3$**)**	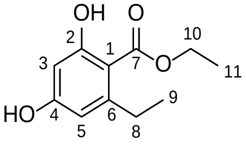 **4 (**$CDCl_3$**)**	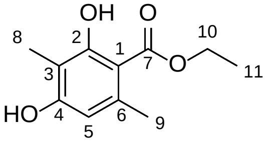 **5 (**$CDCl_3$**)**	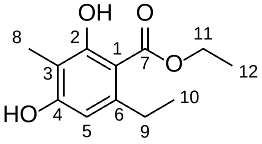 **6 (**$CDCl_3$**)**
Position	δ_H (multi., *J* in Hz)	δ_H (multi., *J* in Hz)	δ_H (multi., *J* in Hz)	δ_H (multi., *J* in Hz)	δ_H (multi., *J* in Hz)	δ_H (multi., *J* in Hz)
3	6.14 (d, 2.5)	–	–	6.25 (d, 2.5)	–	–
5	6.20 (d, 2.5)	6.21 (s)	6.23 (s)	6.29 (d, 2.5)	6.20 (s)	6.22 (s)
8	2.81 (q, 7.4)	2.10 (s)	2.11 (s)	2.90 (q, 7.4)	2.10 (s)	2.10 (s)
9	1.13 (t, 7.4)	2.46 (s)	2.83 (q, 7.4)	1.19 (t, 7.4)	2.47 (s)	2.84 (q, 7.4)
10	3.87 (s)	3.92 (s)	1.15 (t, 7.4)	4.40 (q, 7.1)	4.39 (q, 7.1)	1.16 (t, 7.4)
11	–	–	3.93 (s)	1.42 (t, 7.1)	1.41 (t, 7.1)	4.40 (q, 7.1)
12	–	–	–	–	–	1.41 (t, 7.1)
2-OH	–	12.04 (s)	12.05 (s)	11.83 (s)	12.13 (s)	12.14 (s)
4-OH	–	5.15 (brs)	5.75 (brs)	5.96 (brs)	5.21 (brs)	5.73 (brs)

These data correspond well to those described in the literature: **1** (Sher and Langer 2008), **2** (Schleich et al. 2006), **3** (De Jesus et al. 1987), **4** (Schleich et al. 2006), **5** (Sher and Langer 2008) and **6** (De Jesus et al. 1987).

Table S2. ^{13}C NMR data of **3** (125 MHz in $CDCl_3$)

Compound	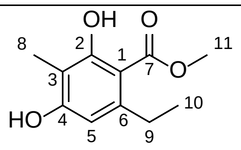 **3**
Positions	δ_C in ppm
1	104.6
2	163.0
3	108.9
4	158.6
5	109.3
6	146.6
7	172.6
8	7.9
9	29.7
10	15.9
11	52.1

These data correspond well to those reported previously (De Jesus et al. 1987).

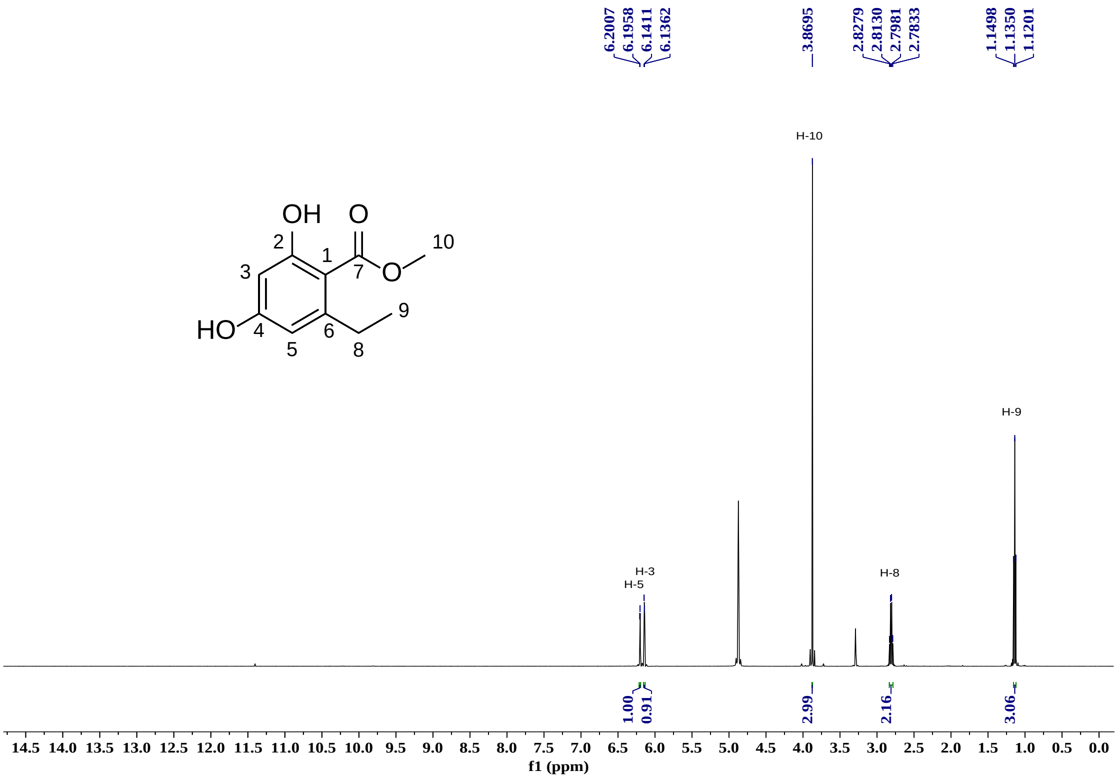

Figure S1. ^{1}H NMR spectrum of compound **1** in CD_3OD (500 MHz)

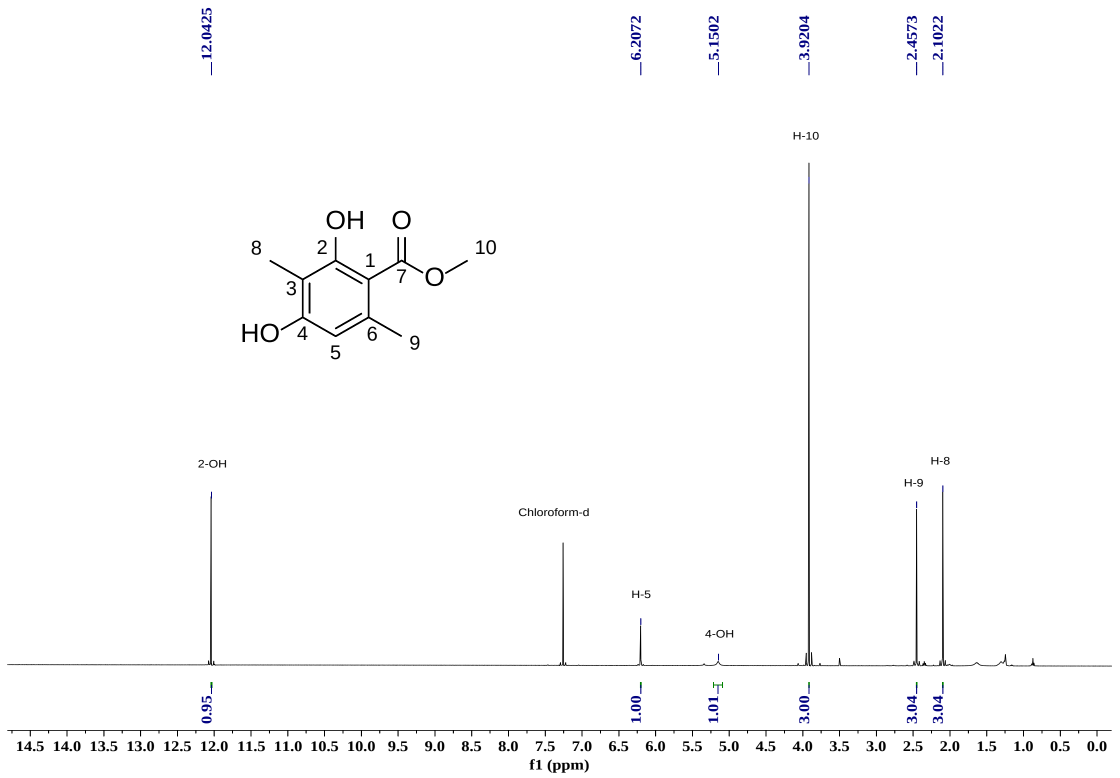

Figure S2. ^{1}H NMR spectrum of compound **2** in $CDCl_3$ (500 MHz)

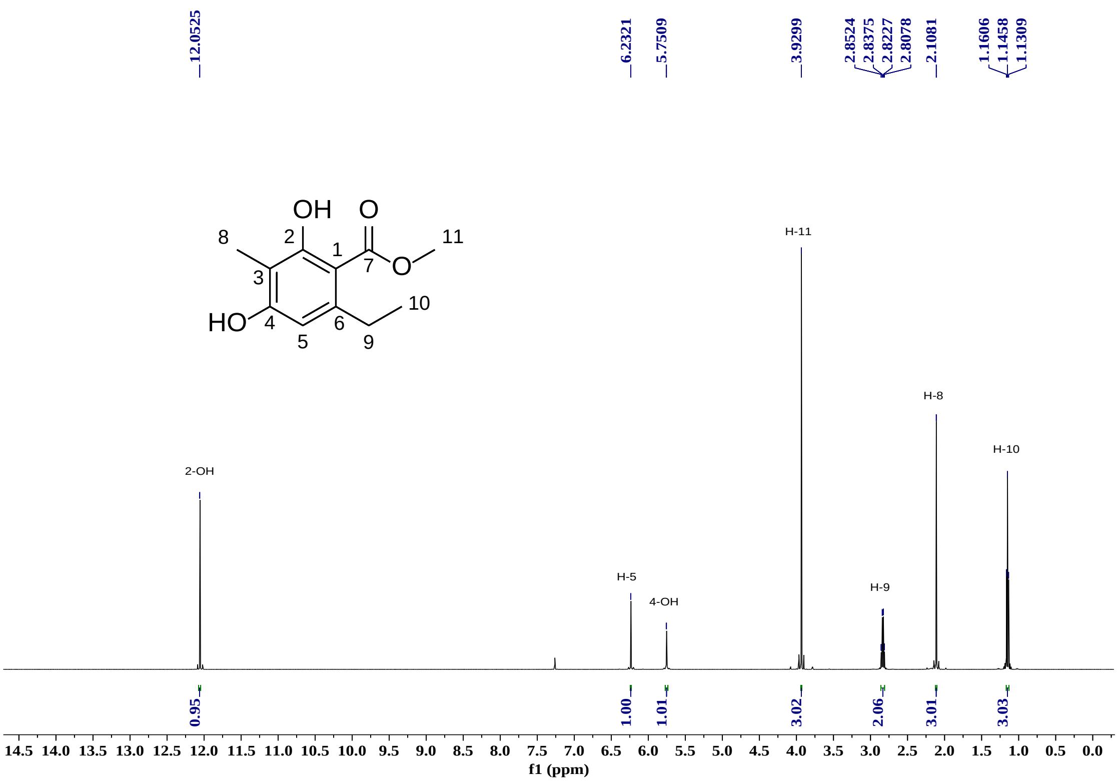

Figure S3. ^{1}H NMR spectrum of compound **3** in $CDCl_3$ (500 MHz)

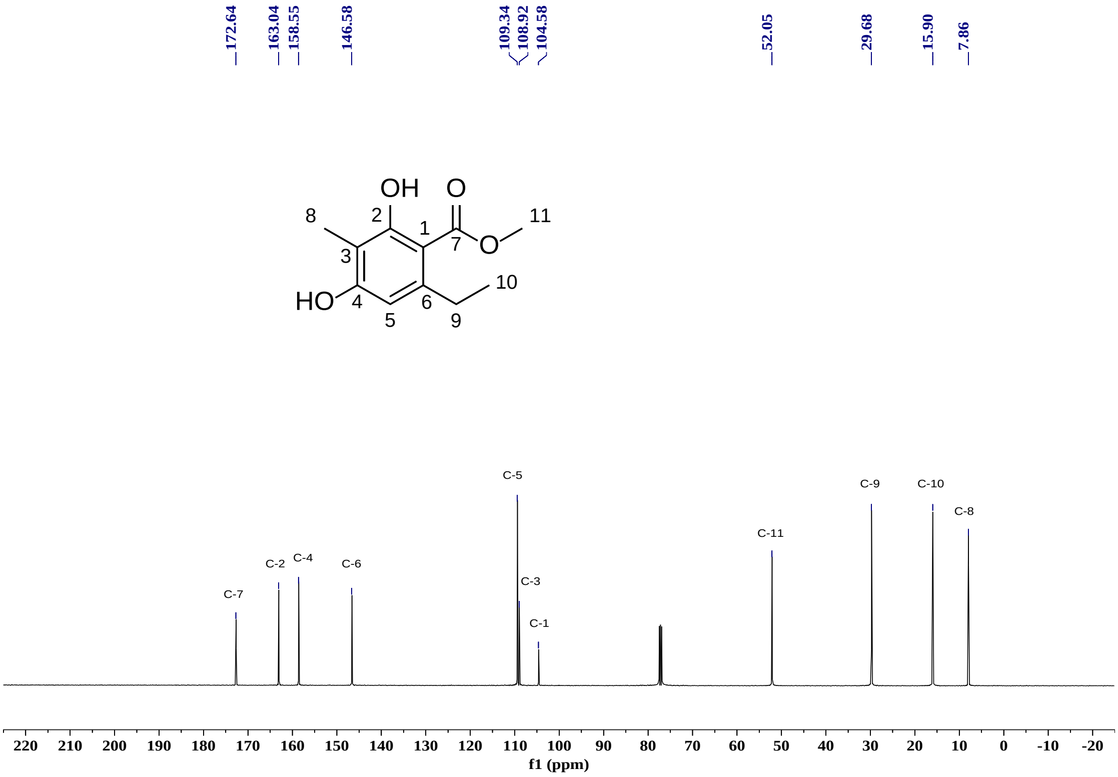

Figure S4. $^{13}C\{^{1}H\}$ NMR spectrum of compound **3** in $CDCl_3$ (125 MHz)

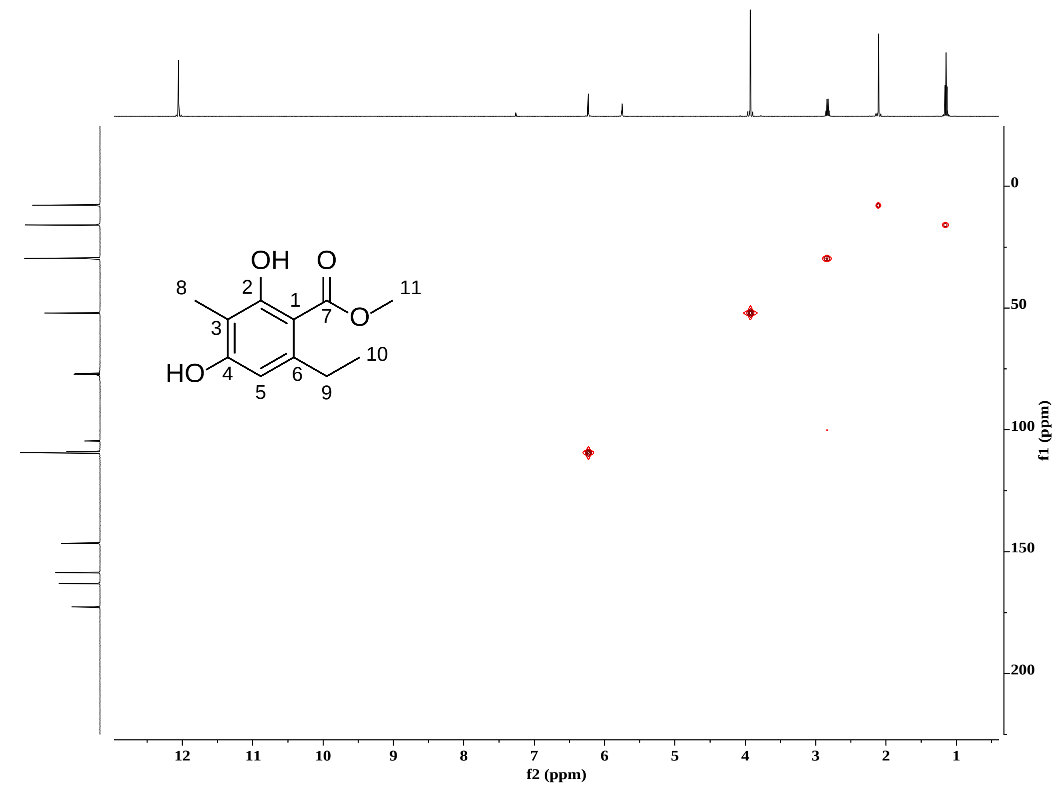

Figure S5. HSQC spectrum of compound **3** in $CDCl_3$

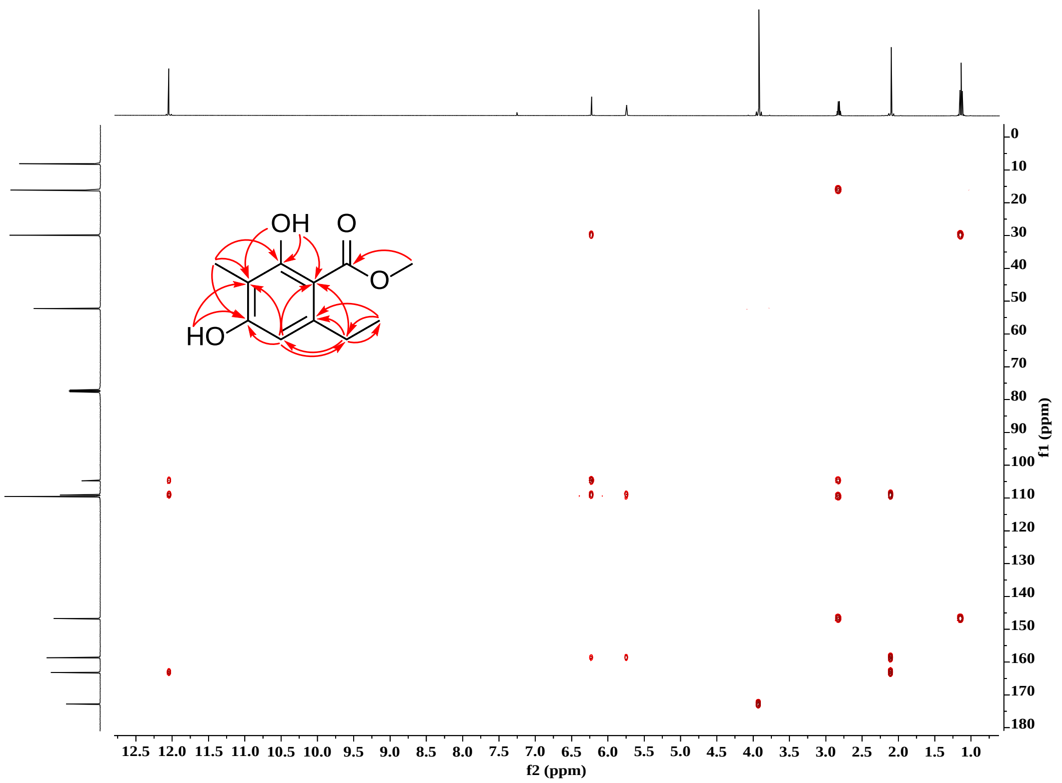

Figure S6. HMBC spectrum of compound **3** in $CDCl_3$

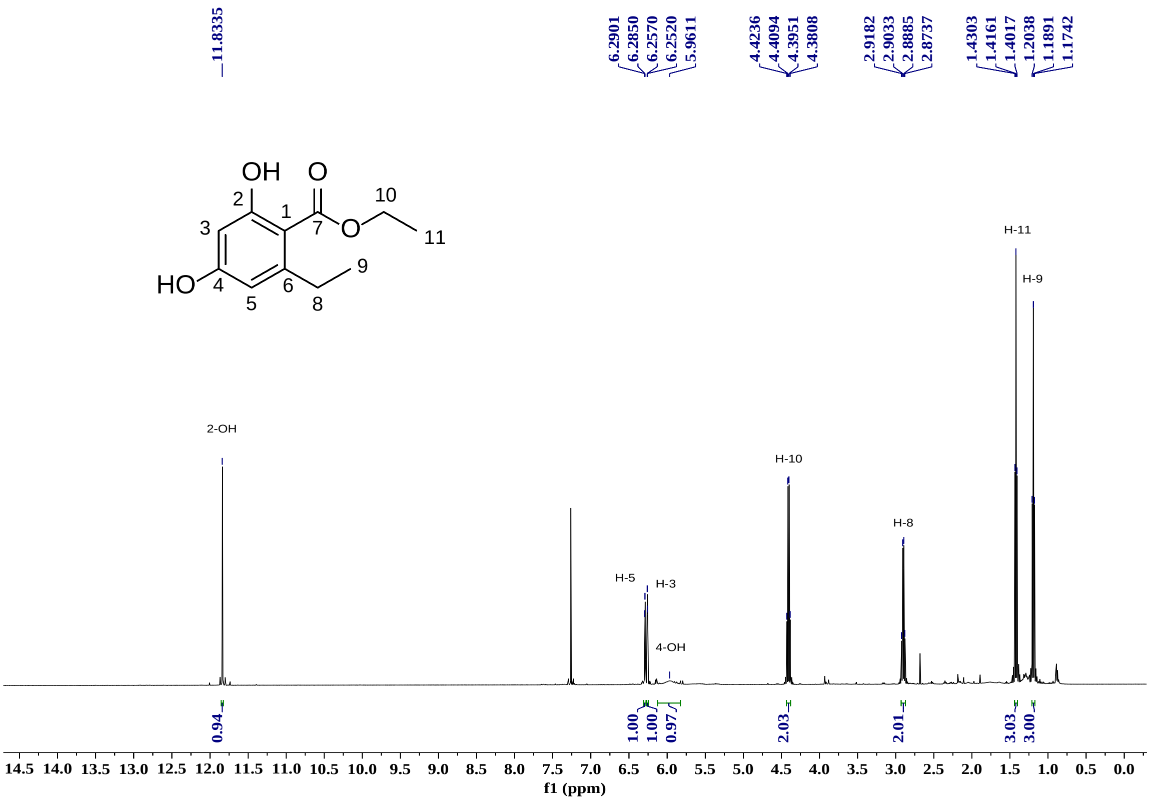

Figure S7. ^{1}H NMR spectrum of compound **4** in $CDCl_3$ (500 MHz)

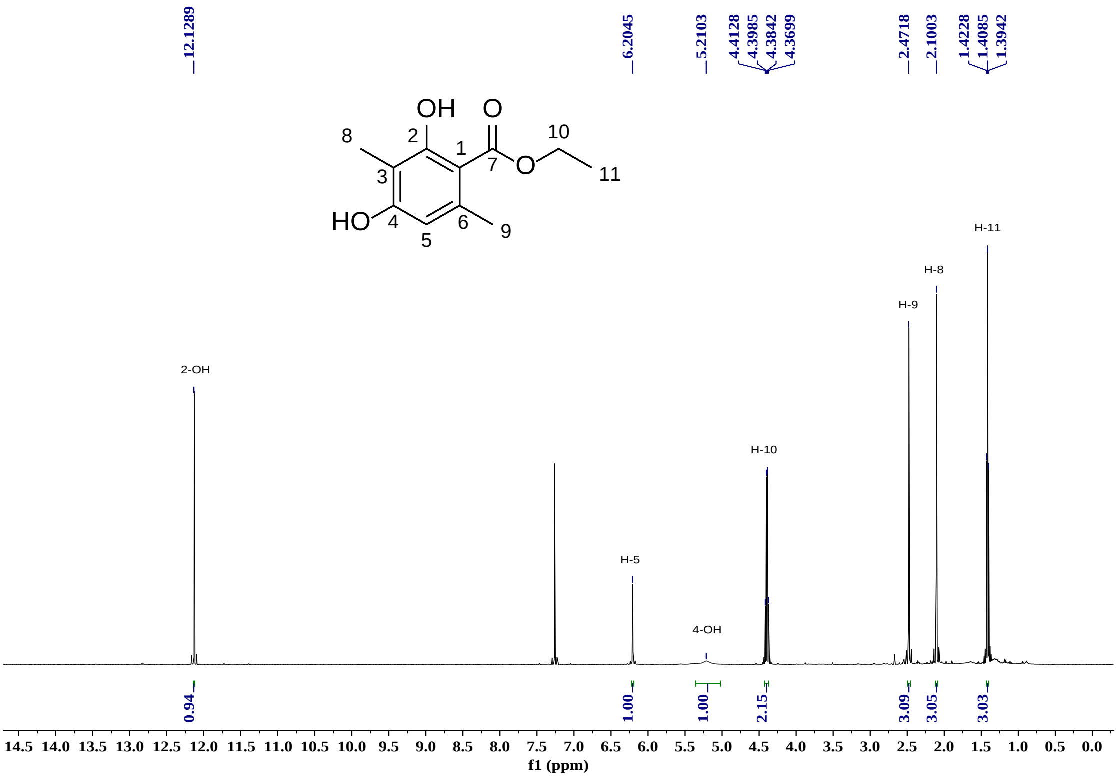

Figure S8. ^{1}H NMR spectrum of compound **5** in $CDCl_3$ (500 MHz)

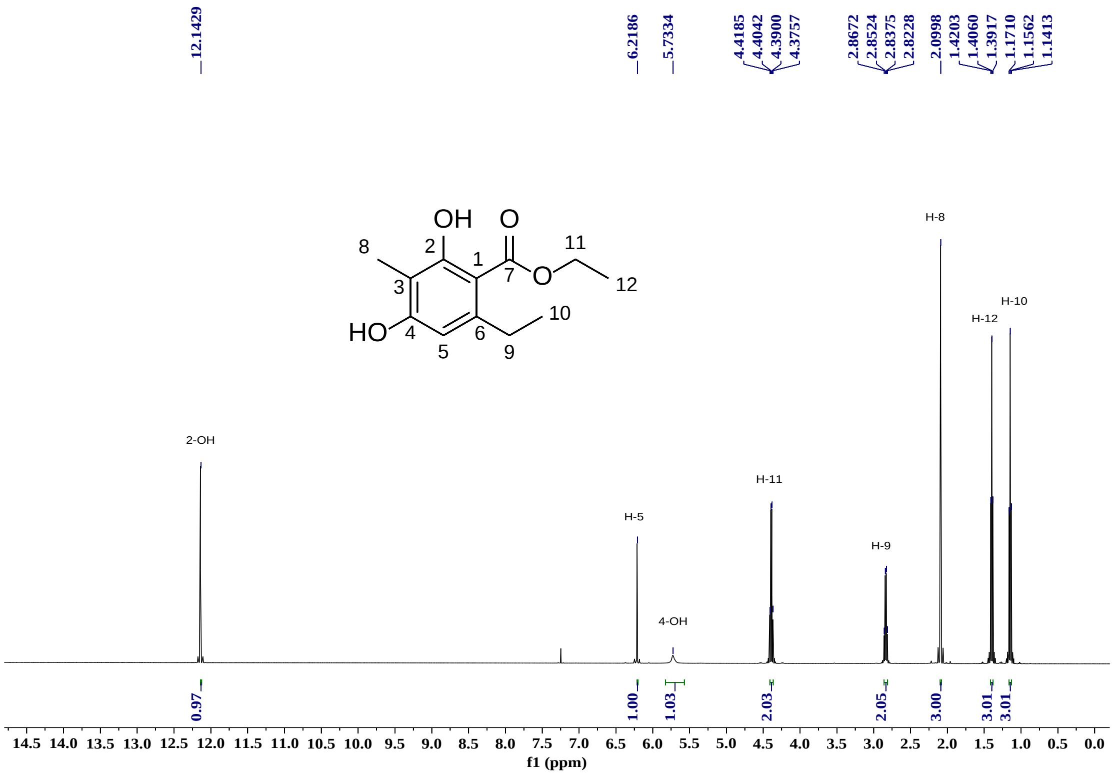

Figure S9. ^{1}H NMR spectrum of compound **6** in $CDCl_3$ (500 MHz)

References

1. De Jesus AE, Horak RM, Steyn PS, Vleggaar R (1987) Metabolites of *Aspergillus ustus*. Part 4. Stable-isotope labelling studies on the biosynthesis of the austalides. J Chem Soc , Perkin Trans 1:2253-2257.

2. Schleich S, Papaioannou M, Baniahmad A, Matusch R (2006) Activity-guided isolation of an antiandrogenic compound of Pygeum africanum. Planta Med 72:547-551.

3. Sher M, Langer P (2008) Regioselective synthesis of functionalized resorcins by cyclization of 1,3-bis(trimethylsilyloxy)-1,3-butadienes with 3,3-dimethoxypentanoyl chloride. Synlett:1050-1052.
